# Inflamed Phylloides Tumour in a Girl: A Challenging Diagnosis in Paediatric Breast Lesions

**DOI:** 10.3390/ijerph15050959

**Published:** 2018-05-11

**Authors:** Ilaria Testa, Cristina Salvatori, Marco Prestipino, Maria Elena Laurenti, Paolo Gerli, Giuseppe Di Cara, Nicola Principi, Susanna Esposito, Mirko Bertozzi

**Affiliations:** 1 Paediatric Clinic, Department of Surgical and Biomedical Sciences, Università degli Studi di Perugia, 06132 Perugia, Italy; ilariatesta@alice.it (I.T.); crisalva_@libero.it (C.S.); giuseppe.dicara@unipg.it (G.D.C.); 2 Pediatric Surgery Unit, S. Maria della Misericordia Hospital, 06132 Perugia, Italy; markprestipino@yahoo.it (M.P.); mirkobertozzi@hotmail.com (M.B.); 3 Section of Pathologic Anatomy and Histology, Department of Experimental Medicine, Università degli Studi di Perugia, 06132 Perugia, Italy; anatomia.patologica@ospedale.perugia.it; 4 S.S. Oncoplastic Surgery-Breast Unit, S. Maria della Misericordia Hospital, 06132 Perugia, Italy; paolo.gerli@ospedale.perugia.it; 5 Università degli Studi di Milano, 20122 Milan, Italy; nicola.principi@unimi.it

**Keywords:** breast abscess, breast tumours, phylloides tumours

## Abstract

*Introduction*: Phylloides tumours (PTs) are rare fibroepithelial neoplasms that account for 0.3–0.9% of all breast tumours. These tumours typically occur in women aged 30–70 years. The occurrence of these tumours in older children and adolescents poses particular diagnostic and therapeutic problems. However, early diagnosis is mandatory because although most of the cases of PTs in children are benign, the borderline and malignant cases with potential negative outcomes cannot be excluded. *Case presentation*: A 12-year-old girl presented at the Paediatric Emergency Department for hyperaemia and warmth of the left breast that occurred a few days prior without fever. The girl experienced menarche 8 months previously. She experienced no previous trauma and she had no family history of breast cancer. On physical examination, the left breast was painful, enlarged and tender. The overlying skin was erythematous and warm. A breast ultrasonography (US) revealed a large mass with features of an abscess, including a hyperechoic wall, scattered internal echoes and hypoechoic peripheral lacunae of apparent colliquative nature. After 4 days of unsuccessful antibiotic therapy, surgical drainage was performed due to the suspicion of a mammary abscess. At the surgical incision site, the lesion was not-well circumscribed and lacked a capsule. In addition, purulent material was not detected. Histological examination revealed that the tissue alterations were compatible with benign PT. With this diagnosis, the girl underwent definitive surgical removal of the lesion. The postoperative period passed without negative events. An US performed 6 months later revealed that no new mass was present at this time, suggesting no recurrence of the tumour. *Conclusion*: This case shows that in the presence of a clinical picture suggesting the inflammation of the breast in adolescent females, PT should be considered as a possible diagnosis and US-guided core biopsy should be considered to confirm this suspicion. Thereafter, when surgical excision is performed, particular attention must be paid to both the preservation of all the normal breast parenchyma and future aesthetic problems.

## 1. Introduction

Phylloides tumours (PTs) are rare fibroepithelial neoplasms that account for 0.3–0.9% of all breast tumours [1]. These tumours typically occur in women aged 30–70 years. However, these tumours have been described in pre- and post-pubertal girls and in men. In men, these tumours have been consistently associated with gynaecomastia [2]. The occurrence of these tumours in older children and adolescents poses particular diagnostic and therapeutic problems. PTs must be differentiated not only from the other neoplastic masses that can appear at this age but also from the variations of breast development, which are typically related to puberty [3].

Moreover, to make their identification even more difficult, PTs can have differing features at presentation. Although generally unilateral, PTs can be bilateral and multifocal. In some cases, the initial signs and symptoms are different from those typically described. Instead of a painless palpable slow-growing mass, PT can present as a rapid accelerating mass in some cases. This can be associated with significant painful hyperaemia and warmth of the overlying breast skin, mimicking breast abscess or mastitis, or with nipple discharge [4,5,6,7,8]. This feature can delay the diagnosis and lead to the use of unjustified diagnostic and therapeutic approaches. However, early diagnosis is mandatory because although most of the cases of PTs in children are benign, the borderline and malignant cases with potential negative outcomes cannot be excluded.

The main aim of this report is to describe the case of a 12-year-old girl with PT in order to remind physicians how PT can be diagnosed and treated when it occurs in children. 

## 2. Case Presentation

A 12-year-old girl presented at the Paediatric Emergency Department of Perugia Hospital due to hyperaemia and warmth of her left breast that had appeared a few days before without fever. Clinical history revealed occurrence of menarche 8 months before and asymmetric thelarche with slight predominance of the left breast 3 months before, although there were no signs of inflammation. No previous trauma was reported.

**Figure 1:**
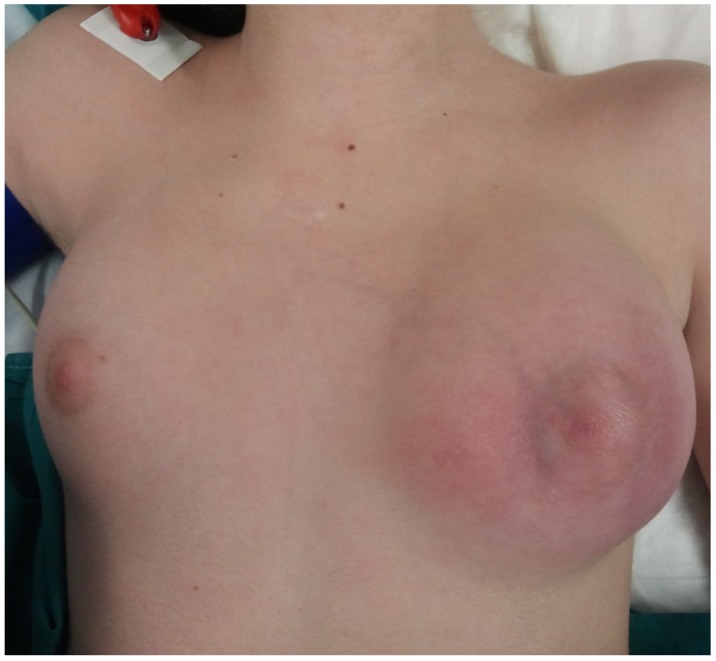
Left breast of a 12-year-old girl: it was painful, enlarged and tender with the overlying skin that was erythematous and warm.

On physical examination, the left breast was painful, enlarged and tender. The overlying skin was erythematous and warm (Figure 1). No palpable axillary lymphadenopathy was detected.

**Figure 2:**
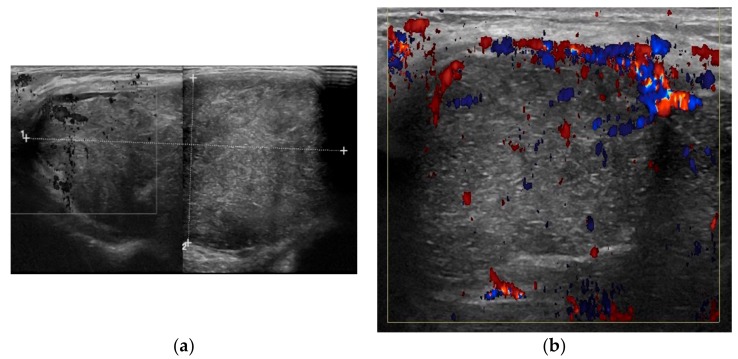
(**a**) Breast ultrasound (US) shows hyperechoic wall; scattered internal echoes and hypoechoic peripheral lacunae of colliquative nature; and (**b**) Doppler US demonstrated increased peripheral and intralesional vascularity with axillary reactive lymphadenopathy.

A breast ultrasonography (US) was performed, revealing a large mass with the features of an abscess, including a hyperechoic wall, scattered internal echoes and hypoechoic peripheral lacunae of apparent colliquative nature (Figure 2a). The Doppler US revealed increased peripheral and intralesional vascularity with axillary reactive lymphadenopathy (Figure 2b). 

Antibiotic therapy with intravenous meropenem (1 g every 8 h) was administered without any significant clinical effect after 4 days.

**Figure 3:**
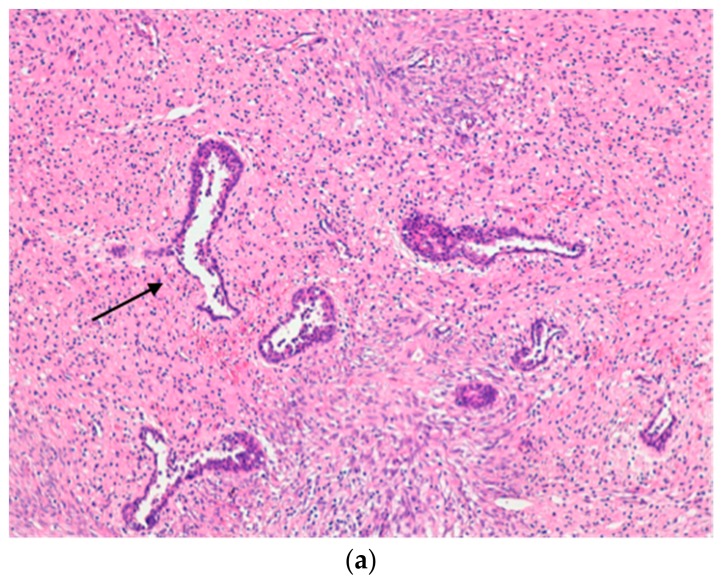

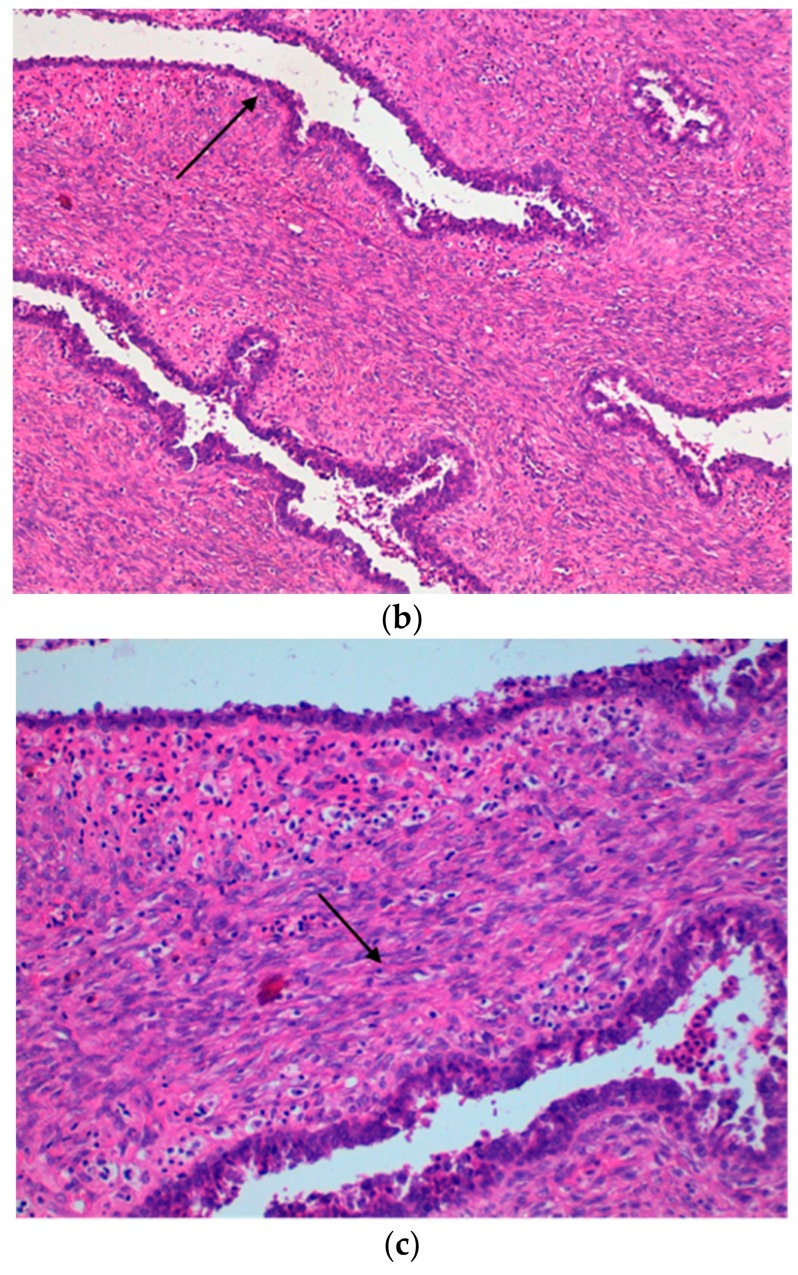
Histological findings in the study patient. (**a**,**b**) (Haematoxylin-Eosin, 100×): different fields of the lesion showing the pericanalicular pattern of the epithelial component (arrow) and stromal cellularity ranging from mild (**a**) to discrete (**b**). On closer examination ((**c**), 200×), the stromal fibroblasts are more densely packed near the epithelial component (arrowhead), with sparse intermingled lymphocytes. No mitosis was documented in 50 high power fields.

Thus, due to the suspicion of a breast abscess, surgical drainage was performed. At the surgical incision site, the mass was not-well circumscribed and lacked a capsule. In addition, purulent material was not detected, suggesting a non-infectious origin. Laboratory tests (white blood cell count, C-reactive protein and procalcitonin values) were in the normal range, which confirmed this supposition. Mass histology revealed tissue alterations compatible with benign PT. This includes the pericanalicular pattern of the epithelial component and stromal fibroblasts that were more densely packed near the epithelial component, with sparse intermingled lymphocytes and no mitosis (Figure 3a–c).

With this diagnosis, the girl underwent definitive surgical removal of the lesion. The postoperative period passed without negative events. An US performed 6 months later revealed that no new mass was present at the time, suggesting no recurrence of the tumour.

The Ethics Committee of Perugia hospital approved the management of this case and the publication of this article. The patient’s parents signed a written informed consent and the patient signed a written assent.

## 3. Discussion

PTs can be classified as benign, borderline and malignant based on histologic findings [9]. According to the World Health Classification (WHO) classifications [10], malignant PTs exhibit marked stromal hypercellularity and cellular pleomorphism, ≥10 mitosis/10 high power fields, stromal overgrowth and infiltrative margins. The cases with moderate or minimal/no alterations are defined as borderline or benign PT, respectively. However, given the lack of defined criteria or clear cut-offs for most of the histologic parameters, histologic findings are not consistently predictive of clinical behaviour in adult patients and malignant transformation has been reported in up to 30% of patients [1].

Fortunately, paediatric PTs are generally benign. During 1973–2004, only 29 cases of malignant PT were reported in the USA [11]. However, malignant transformation cannot be excluded. Multiple conditions, such as osteosarcoma, neurofibromatosis and lymphoma, can predispose the child to malignant PT [12,13,14]. The same findings can be true for the patients with genetic mutations associated with an increased risk of cancer [15]. Consequently, the early diagnosis of PT is mandatory to avoid unnecessary diagnostic therapeutic approaches and ensure the complete surgical removal of the tumour. Unfortunately, the identification of paediatric PT is difficult, particularly when the initial signs are atypical. Cutaneous structures can be compressed by the tumour mass and skin inflammation can develop as reported in this case [3]. The infarction of the tumour can lead to nipple discharge [4]. Moreover, some pubertal developmental breast lesions causing or mimicking a mass can complicate the diagnostic approach [16]. Premature thelarche, asymmetric development of breast buds, supernumerary breast tissue and gynaecomastia are the most common prepubertal and peripubertal problems. Post-pubertal variations of development include mammary duct ectasia, cystic breast changes and infectious problems, such as mastitis and breast abscess. Moreover, a number of neoplastic processes can be confused with PT and delay diagnosis, with juvenile fibroadenoma being the most common [17]. Juvenile fibroadenoma comprises 1–8% of breast lesions in the adolescent population. Although juvenile fibroadenoma is easily distinguishable from PT from a histological point of view, the condition can initially cause signs and symptoms that are similar to those of PT. Moreover, the US features are quite similar [17] and do not permit either the differentiation of PT from juvenile fibroadenoma or the evaluation of the type of PT [18]. Only magnetic resonance imaging (MRI) can be useful for the diagnosis and to establish the relationship of the mass with the surrounding normal tissue [19].

In our case, the situation of the apparent mammary abscess misled doctors and surgical drainage was performed. However, in presence of a defined lump in the left breast, a US-guided core biopsy is the appropriate approach to reveal the presence of a biphasic lesion [20]. With that result, a surgical excision of the entire lump and a small normal tissue margin instead of a surgical drainage with an open biopsy of the mass is recommended [20].

## 4. Conclusions

This case shows that in the presence of a clinical picture suggesting inflammation of the breast in adolescent females, PT should be considered as a possible diagnosis. The apparent mammary abscess could mislead doctors and US-guided core biopsy should be considered to confirm this suspicion as the first diagnostic approach. Thereafter, when the PT diagnosis is confirmed and the surgical excision is performed, particular attention must be paid to both the preservation of all the normal breast parenchyma and future aesthetic problems.
